# Respiratory Syncytial Virus (RSV)-Induced Autoimmune Hemolytic Anemia Presenting as Syncope: A Case Report

**DOI:** 10.7759/cureus.77369

**Published:** 2025-01-13

**Authors:** James T Johns

**Affiliations:** 1 Emergency Medicine, University Hospitals St. John Medical Center, Westlake, USA

**Keywords:** autoimmune hemolytic anemia (aiha), community emergency department, emergency medicine, respiratory syncytial virus (rsv), unusual cause of recurrent syncope

## Abstract

Autoimmune hemolytic anemia (AIHA) is a condition that causes an individual’s immune system to destroy its own red blood cells. Immune cells are activated against the red blood cell antigens to induce hemolysis. Patients typically present with symptomatic anemia when the extent of hemolysis overcomes the bone marrow’s ability to compensate. Steroids are typically the first-line treatment, along with supportive care and blood transfusion for severe anemia. This case report details a patient who had presented to the ED after a syncopal episode that was ultimately due to respiratory syncytial virus (RSV)-induced warm AIHA.

## Introduction

Autoimmune hemolytic anemia (AIHA) is a condition that causes an individual’s immune system to destroy its own red blood cells. Immune cells are activated against the red blood cell antigens to induce hemolysis [1]. AIHA can be broadly classified as a warm or cold-mediated disease. Warm AIHA is more common than cold-mediated disease and occurs at body temperature. Warm AIHA is primarily IgG mediated, where hemolysis occurs extravascularly [2]. AIHA may be either primary (idiopathic) or secondary. Primary disease occurs when no disease process is clearly associated with the hemolytic anemia (Scheckel). Secondary causes of AIHA are varied, but include conditions such as hematologic malignancies, drugs, autoimmune disorders, solid tumors, and infectious diseases [3, 4]. This case report details a patient who had presented to the ED after a syncopal episode that was ultimately due to respiratory syncytial virus (RSV)-induced warm AIHA.

## Case presentation

A 25-year-old female with a past medical history of asthma and depression presented to the emergency department (ED) for syncope. The patient was evaluated at the urgent care for upper respiratory tract infection (URI) symptoms. While being evaluated at the urgent care, the patient had a syncopal episode. She fell off the examination table and hit her head. She was complaining of a headache currently. The patient was not on anticoagulation. The patient denied any additional injuries secondary to the fall. The patient had been having four days of non-productive cough, rhinorrhea, odynophagia, chest pain, shortness of breath, abdominal pain, nausea and vomiting, and subjective fever. The patient denied any blood in the vomit or coffee ground emesis. The patient denied any blood in the stools or melanotic stools. On arrival to the ED, the patient was febrile (T 39.9°C), tachycardia (110 beats per minute), normal respiratory rate (16), normotensive (132/68), and was oxygenating well on room air. The patient’s airway was intact, and she was speaking in full sentences. The patient had no respiratory distress or increased work of breathing and had equal bilateral breath sounds with wheezing present. The patient had strong, equal radial pulses. The patient was resting comfortably on the bed in no acute distress. On physical examination, the patient was noted to have a cephalohematoma of the right parietal scalp with no palpable skull fracture and no clinical signs of basilar skull fracture. No other traumatic injuries were noted on examination. The patient had a normal neurologic examination. The patient was pale and tachycardic. The remainder of her physical examination was normal.

Given the patient’s syncope and abnormal vital signs, further laboratory and radiologic workup was pursued. An electrocardiogram (ECG), complete blood count (CBC) with automatic differential, comprehensive metabolic panel (CMP), venous thromboembolism (VTE) exclusion d-dimer, lactate, troponin, human chorionic gonadotropin (hCG), urinalysis, viral testing (influenza a and b, SARS-COV-2, and RSV) blood cultures, and chest X-ray were ordered. ECG revealed sinus tachycardia with a ventricular rate of 116 beats per minute, normal axis, no acute ischemic changes or injury pattern, normal QT interval, no delta wave, no evidence of Brugada syndrome, and no blocks. RSV was detected via polymerase chain reaction (PCR). CBC demonstrated a normocytic anemia with a hemoglobin of 7.3 g/dL, no leukocytosis, and a normal platelet count. CMP was significant for an elevated total bilirubin of 3.6 mg/dL, normal aspartate transaminase (AST), normal alanine transaminase (ALT), normal alkaline phosphatase, normal blood urea nitrogen (BUN) and creatinine, and normal glucose. High-sensitivity troponin was undetectable. The chest X-ray was normal and showed no evidence of pneumonia, pneumothorax, mediastinal widening, or any other acute abnormalities. D-dimer was minimally elevated at 501 ng/mL, and a pulmonary embolism (PE) computed tomography (CT) angiography of the chest was ordered. The CT angiography of the chest was negative for any evidence of PE, but did show patchy multifocal ground glass and semisolid centrilobular nodular opacities in the left upper lung and the left lower lung likely representing bronchopneumonia with bronchiolitis.

In the setting of new anemia and elevated bilirubin, there was suspicion for hemolysis. Therefore, reticulocytes, lactate dehydrogenase (LDH), haptoglobin, and fractionated bilirubin were added to the laboratory evaluation. Direct bilirubin resulted at 0.5 mg/dL, and indirect bilirubin was elevated at 3.1 mg/dL. The reticulocyte count was elevated at 7.6% and LDH was elevated at 436 U/L; both are consistent with hemolytic anemia. A complete list of laboratory results obtained from the ED is provided in Table 1.

**Table 1:** Emergency Department Laboratory Values eGFR: estimated glomerular filtration rate; ALT: Alanine transaminase; AST: Aspartate transaminase; LDH: Lactate dehydrogenase; INR: International normalized ratio; aPTT: activated partial thromboplastin time; WBC: White blood cells; nRBC: nucleated red blood cells; MCV: Mean corpuscular volume; MCH: Mean corpuscular hemoglobin; MCHC: Mean corpuscular hemoglobin concentration; VTE: Venous thromboembolism.

	Latest Reference Ranges and Units	Result
Glucose	74 - 99 mg/dL	118
Sodium	136 - 145 mmol/L	135
Potassium	3.5 - 5.3 mmol/L	4.0
Chloride	98 - 107 mmol/L	102
Bicarbonate	21 - 32 mmol/L	24
Anion Gap	10 - 20 mmol/L	13
Blood Urea Nitrogen	6 - 23 mg/dL	11
Creatinine	0.50 - 1.05 mg/dL	0.79
EGFR	>60 mL/min/1.73m^2^	>90
Calcium	8.6 - 10.3 mg/dL	8.8
Albumin	3.4 - 5.0 g/dL	4.5
Alkaline Phosphatase	33 - 110 U/L	41
ALT	7 - 45 U/L	38
AST	9 - 39 U/L	38
Bilirubin, Total	0.0 - 1.2 mg/dL	3.6
Bilirubin, Direct	0.0 - 0.3 mg/dL	0.5
Total Protein	6.4 - 8.2 g/dL	7.1
LDH	84 - 246 U/L	436
Lactate	0.4 - 2.0 mmol/L	1.1
Troponin I, High Sensitivity	0 - 13 ng/L	<3
INR	0.9 - 1.1	1.2
Protime	9.8 - 12.8 seconds	13.5
aPTT	27 - 38 seconds	27
D-Dimer, Quantitative VTE Exclusion	<=500 ng/mL FEU	501
WBC	4.4 - 11.3 x 10^3^/uL	6.0
nRBC	0.0 - 0.0 /100 WBCs	1.0
RBC	4.00 - 5.20 x 10^6^/uL	2.55
Hemoglobin	12.0 - 16.0 g/dL	7.3
Hematocrit	36.0 - 46.0%	23.3
MCV	80 - 100 fL	91
MCH	26.0 - 34.0 pg	28.6
MCHC	32.0 - 36.0 g/dL	31.3
Red Cell Distribution Width	11.5 - 14.5%	19.1
Platelets	150 - 450 x 10^3^/uL	243
Neutrophils %	40.0 - 80.0%	68.9
Immature Granulocytes %, Automated	0.0 - 0.9%	1.8
Lymphocytes %	13.0 - 44.0%	18.5
Monocytes %	2.0 - 10.0%	5.3
Eosinophils %	0.0 - 6.0%	5.2
Basophils %	0.0 - 2.0%	0.3
Neutrophils Absolute	1.20 - 7.70 x 10^3^/uL	4.12
Immature Granulocytes Absolute, Automated	0.00 - 0.70 x 10^3^/uL	0.11
Lymphocytes Absolute	1.20 - 4.80 x 10^3^/uL	1.11
Monocytes Absolute	0.10 - 1.00 x 10^3^/uL	0.32
Eosinophils Absolute	0.00 - 0.70 x 10^3^/uL	0.31
Basophils Absolute	0.00 - 0.10 x 10^3^/uL	0.02
Immature Reticulocyte fraction	<=16.0%	40.3
Reticulocyte Absolute	0.018 - 0.083 x 10^6^/uL	0.190
Reticulocyte %	0.5 - 2.0%	7.6
Reticulocyte Hemoglobin	28 - 38 pg	29

In the Emergency Department, the patient’s fever was treated with 650 mg acetaminophen. She was ordered 1,000 mL lactated ringer’s bolus because of her tachycardia and syncope. The patient was administered ipratropium-albuterol nebulized solution and 125 mg methylprednisolone sodium succinate to treat her asthma exacerbation. 1 unit of packed red blood cells was ordered to be transfused in the setting of new anemia and syncope.

Hematology was consulted from the ED. The hematologist recommended admission to the hospital and intravenous (IV) steroids. Steroids had previously been given earlier in the patient’s ED course.

On day 1 of hospitalization, hemoglobin decreased to 6.1 g/dL. There had been a delay in the transfusion of packed red blood cells secondary to the patient’s antibodies and the need to obtain blood products from the Red Cross. She was given the blood transfusion on day 1 of hospitalization. 150 mg methylprednisolone IV daily was continued. The patient was also started on ceftriaxone and azithromycin to treat the bronchopneumonia that was noted on her CT scan. Hematology ordered direct antiglobulin test (DAT), DAT IgG, and DAT complement. The DAT was positive, DAT IgG was positive, and DAT complement was negative, all consistent with warm hemolytic anemia.

On day 2 of hospitalization, the hemoglobin decreased to 5.7 g/dL, and an additional 2 units of packed red blood cells were transfused. The IV steroids were continued. After the transfusion of blood products, hemoglobin improved to 8.1 g/dL. On day 3 of hospitalization, hemoglobin remained stable. The patient was transitioned to oral steroids, prednisone 40 mg twice a day. The patient was discharged on day 3 of hospitalization with outpatient hematology follow-up.

The patient followed up with the hematologist outpatient two weeks after discharge and had been off prednisone for one week. Hemoglobin had decreased to 6.8 g/dL and an outpatient transfusion of 1 unit of packed red blood cells was arranged followed by starting rituximab. The patient did not tolerate the rituximab infusion, and it was discontinued early. On follow-up three weeks after discharge, the patient’s hemoglobin had improved to 8.6 g/dL. One week later, four weeks after discharge, the patient’s hemoglobin continued to improve, and the patient had no further signs or symptoms of hemolysis.

## Discussion

Syncope accounts for 1% - 1.5% of all ED visits [5]. A majority of syncopal events are secondary to either vasovagal syncope or orthostatic hypotension, comprising approximately 60% of patients presenting to the Emergency Department. The 30-day mortality rate for patients presenting to the ED is 0.7% [6]. While the 30-day mortality rate may be low, it is important to identify those patients who are at higher risk and require further workup and/or hospital admission. To risk stratify patients, several clinical decision tools have been created. The San Francisco Syncope Rule, Evaluation of Guidelines in Syncope Study, and Osservatorio Epidemiologico sulla Sincope nel Lazio have been externally validated. However, not one scoring system has been found to be superior to the others [7].

Several high-risk features have been identified which should prompt further investigation or admission. These include ECG abnormalities, severe cardiac structural or coronary artery disease, hypotension, anemia or evidence of acute hemorrhage, syncope during marked exertion, family history of sudden cardiac death, or palpitations at the time of loss of consciousness [8]. Abnormal ECG is broad, and each clinical decision tool has a different definition. However, an ECG with bradyarrhythmia, tachyarrhythmia, conduction defects (as noted with QRS widening and bundle branch blocks), preexcitation, QT prolongation, ST segment changes, T wave inversions, and evidence of electrolyte channelopathies (i.e. Brugada syndrome) are consistent with a possible cardiac etiology of syncope [7,9]. Vasovagal syncope often has preceding symptoms for minutes before the loss of consciousness. The lack of prodromal symptoms would likely indicate a diagnosis other than vasovagal syncope [10] and therefore should also be considered a high-risk clinical feature.

This case features a patient with a syncopal episode secondary to acute blood loss from hemolytic anemia. Autoimmune hemolytic anemia may be either primary or secondary to an underlying health condition that leads to immune system dysfunction [1]. Viral infections and other infectious diseases are a known secondary cause of autoimmune hemolytic anemia [2]. There have been very few case reports identifying RSV as the secondary cause of hemolytic anemia, all of which have been paroxysmal cold hemoglobinuria [11, 12]. Abidoye et al. described a case of warm autoimmune hemolytic anemia that was secondary to Epstein-Barr Virus, another viral infection that is commonly associated with cold autoimmune hemolytic anemia [13]. This author was unable to find any other case studies in the literature on RSV as the secondary cause of warm autoimmune hemolytic anemia. Warm autoimmune hemolytic anemia occurs from the destruction of red blood cells by immunoglobulin G (IgG) autoantibodies. The mainstays of treatment have been steroids, splenectomy, and immunosuppressants with rituximab being used in steroid-refractory cases [3].

## Conclusions

This was a patient who presented to the Emergency Department with syncope and was found to have significant anemia secondary to warm autoimmune hemolytic anemia. The AIHA was most likely secondary to the patient’s RSV infection. Syncope is a common presenting complaint to the Emergency Department and is most often secondary to a benign cause. However, the Emergency Medicine provider must be able to identify which patients are at higher risk of short-term morbidity and mortality which can often be elucidated from a thorough history and physical examination. Those patients deemed to represent a higher risk should have a complete evaluation including laboratory and radiologic testing and occasionally hospital admission for further management.
